# Elevated LDH greater than 400 U/L portends poorer overall survival in diffuse large B-cell lymphoma patients treated with CD19 CAR-T cell therapy in a real world multi-ethnic cohort

**DOI:** 10.1186/s40164-021-00248-9

**Published:** 2021-12-09

**Authors:** Emma Rabinovich, Kith Pradhan, R. Alejandro Sica, Lizamarie Bachier-Rodriguez, Ioannis Mantzaris, Noah Kornblum, Aditi Shastri, Kira Gritsman, Mendel Goldfinger, Amit Verma, Ira Braunschweig

**Affiliations:** Department of Oncology, Montefiore Medical Center, Bronx, NY 10461 USA

**Keywords:** CAR T-cell therapy, DLBCL, LDH

## Abstract

Anti-CD19 chimeric antigen receptor T-cell therapies have shown striking clinical activity in diffuse large B-cell lymphoma but robust biomarkers predictive of responsiveness are still needed. We treated a multi-ethnic cohort of 31 diffuse large B-cell lymphoma patients with axicabtagene ciloleucel with an overall response rate of 71%. Analysis of various biomarkers identified a significant decrease in overall survival with elevated lactate dehydrogenase, measured both at time of cell infusion and before lymphodepletion. Lactate dehydrogenase was prognostic in a multivariate analysis [HR = 1.47 (1.1–2.0)] and a value of 400 U/L at time of infusion and a value of 440 U/L before lymphodepletion provided the best prognostic cutoffs for overall survival in our cohort. These data demonstrate efficacy of anti-CD19 chimeric antigen receptor T-cell therapy in a diverse inner city population and demonstrate novel lactate dehydrogenase cutoffs as prognostic biomarkers.

## To the Editor

Treatment of relapsed and refractory diffuse large B-cell lymphoma (DLBCL) has long been a challenge fraught with poor outcomes, prompting the search for novel treatment options [1]. Anti-CD19 chimeric antigen receptor (CAR) T-cell therapies have shown striking clinical activity in relapsed and refractory diffuse large B-cell lymphoma (DLBCL) with response rates of 40–50% in clinical trials [2–6]. Wider use of these therapies have exposed some notable concerns regarding treatment-related toxicity, chiefly cytokine release syndrome; manufacturing capacity; and relapse rates [7, 8]. Due to the high morbidity and financial costs associated with these therapies, it is important to identify robust biomarkers predictive of responsiveness or resistance to treatment. This is especially true when treating diverse real-world patient populations not well represented in clinical trials.

We identified 31 consecutive patients who underwent CAR T-cell therapy with axicabtagene ciloleucel between 6/2018 and 12/2020, all with late stage DLBCL and median age of 64 years. Of these, 22 achieved either partial response (n = 2, 6.5%) or complete response (n = 20, 64.5%) at an overall median follow up time of 155 days (range 11–876 days). Five of those that achieved a response had a subsequent relapse of disease. Seven were deceased at the conclusion of data collection in January 2021. Our multi-ethnic cohort included 14 (45%) Caucasian, 10 (32%) Hispanic and 5 (16%) African American patients.

Biomarkers evaluated in this analysis included demographics, immunohistochemistry, treatment history, performance status, international prognostic index scoring, lactate dehydrogenase (LDH) at different points during treatment, and toxicity scoring. LDH measurements were collected at disease relapse (median 263U/L; range 123–1552), before lymphodepletion (median 327 U/L; range 120–1277), and at time of cell infusion (median 237 U/L; range 128–1248). Our analysis identified a statistically significant difference in overall survival (OS) only with LDH at time of cell infusion (p value 0.00324) and LDH before lymphodepletion (p value 0.00085). In our cohort, every 100 U/L rise in LDH at time of cell infusion corresponded to 34% higher risk of death with hazard ratio of 1.34 (range 1.10, 1.64). Likewise, every 100 U/L rise in LDH before lymphodepletion corresponded to 40% higher risk of death with hazard ratio of 1.40 (range 1.15, 1.71).

When we accounted for age, race, ethnicity, and gender in the multivariate analysis of LDH at cell infusion, the difference in OS remained significant (p value 0.018). After accounting for these covariates in our cohort, every 100 U/L rise in LDH at cell infusion corresponded to a 47% higher risk of death, hazard ratio of 1.47 (range 1.07, 2.03). Table 1 displays results of the univariate analysis across all 21 variables. Row ‘LDH/100 (U/L)’ corresponds to LDH divided by 100 and provides hazard ratio confidence intervals of each 100 unit increase in LDH. Multivariate analysis of LDH before lymphodepletion accounting for these same factors confirmed the difference in OS remained significant (p value 0.015) here as well, with hazard ratio of 2.11 (range 1.15, 3.85).

**Table 1:** Analysis across 21 assessed variables

		# of patients	% of patients	Hazard ratio for overall survival (range)	P-value
Gender	Female	12	39	1	
	Male	19	61	1.01 (0.24–4.30)	0.984
Race, ethnicity	White, non-Hispanic	14	45	1	
	White, Hispanic	8	26	1.65 (0.22–11.9)	0.372
	Black, non-Hispanic	5	16	2.90 (0.40–20.9)	0.53
	Black, Hispanic	2	6	3.08 (0.26–36.4)	0.29
	Not specified	2	6		
ECOG performance status	0–1	20	65	1	
	2	11	35	0.60 (0.12–3.01)	0.54
R-IPI	Very good or good	13	42	1	
	Poor	18	58	6.00 (0.74–48.9)	0.094
Disease stage	3	2	6	1	
	4	29	94	High	0.999
Cell of origin	Non-GCB	10	32	1	
	GCB	10	32	1.41 (0.31–6.40)	0.295
	Not specified	11	35		
Bulky disease	Yes	15	48	1.79 (0.42–7.55)	0.426
Double expressor	Yes	9	29	2.02 (0.48–8.49)	0.338
Triple expressor	Yes	4	13	0.297 (0.036–2.44)	0.259
CNS involvement prior therapy	Yes	2	6	1.26 (0–inf)	0.999
Prior therapy with RCHOP	Yes	29	94	High	0.999
Prior autologous HSCT	Yes	12	39	0.34 (0.06–1.80)	0.203
Age (c)	29–84	31	100	1.01 (0.956–1.08)	0.623
Ki67% (c)	40–99	29	94	1.02 (0.96–1.08)	0.499
	Not specified	2	6		
LDH at cell infusion (U/L) (c)	128–1248	31	100	1.00 (1.00–1.00)	0.003
LDH at cell infusion/100 (U/L) (c)	128–1248/100	31	100	1.34 (1.10–1.64)	0.003
LDH before lymphodepletion (U/L) (c)	120–1277	31	100	1.41 (1.15–1.72)	0.0008
LDH at disease recurrence (c)	123–1552	31	100	1.09 (0.92–1.27)	0.325
Number of metastatic sites (c)	0–6	31	100	1.18 (0.713–1.95)	0.522
CRS (c)	0–3	31	100	0.94 (0.38–2.32)	0.891
ICANS (c)	0–4	31	100	1.19 (0.71–2.00)	0.511
CARTOX (c)	0–10	31	100	0.88 (0.74–1.04)	0.140
Tocilizumab doses (c)	0–4	31	100	1.04 (0.58–1.88)	0.886

Variables (c) were treated as continuous; remainder as categorical

**Fig. 1:**
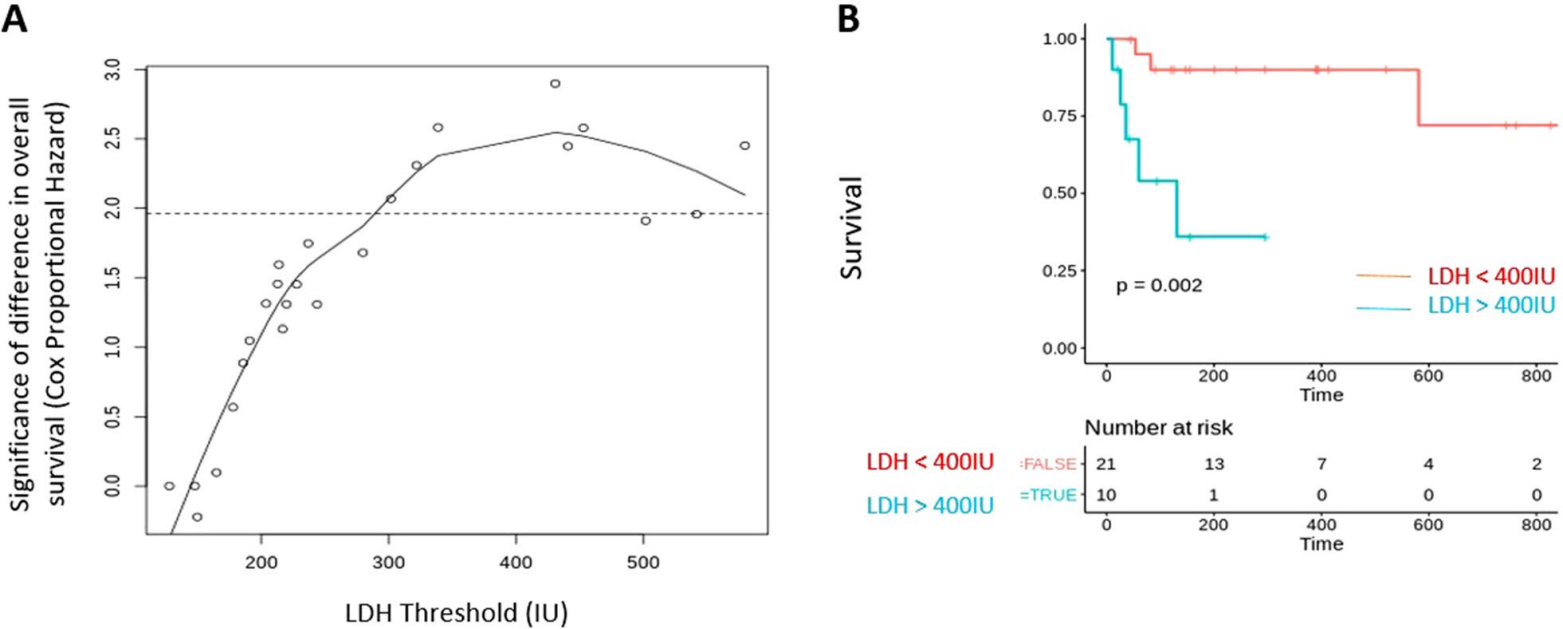
LDH is an adverse prognostic variable in DLBCL patients receiving CAR T-cell therapy. **A** Correlation of LDH levels at the time of cell infusion and significance for difference in overall survival between the LDH cutoffs. A LDH cutoff level of 400 IU shows the highest difference in survival between patients. **B** Overall survival at LDH levels above and below 400 IU as shown by Kaplan Meier curves

Correlation of LDH levels at cell infusion revealed that a value of 400 U/L was associated with maximal prognostic significance for OS (Fig. 1A). OS for patients with LDH greater than 400 U/L at time of CAR T-cell infusion was significantly lower than that of patients with LDH less than 400 U/L at time of diagnosis (Median survival not reached vs 131 days; p 0.002) (Fig. 1B). Similar analysis for LDH levels prior to lymphodepletion yielded a threshold of 440 U/L. Our findings of decreased OS in patients with high LDH appear, on our analysis, to be unrelated to disease relapse, and correspond to disease progression despite therapy and therapy-related toxicity.

A high LDH is a potential marker of greater burden of more aggressive disease and has been evaluated in previous studies. Multivariate analysis of clinical trial data for tisagenlecleucel was first to suggest that patients with elevated pre-infusion LDH had poorer performance free survival and OS [9]. Larger scale analysis from the US Lymphoma CAR T Consortium evaluating outcomes with axicabtagene ciloleucel also found higher LDH before conditioning to be a significant predictor of lower OS on univariate and multivariate analysis [10]. A French study looking at outcomes across five lymphoma centers for patients treated with either therapy had similar findings [11]. Our study defines a LDH of 400 IU as a novel cutoff for poor prognosis.

Though our study represents a single center analysis with relatively small sample size, it offers a real-world perspective from a diverse patient population treated only as recently as the last 2–3 years. The population includes all tumor subtypes with variable prognostic features. Black and Hispanic patients comprised nearly half (n = 15, 48%) of all patients with no significant difference in OS in either population, despite prior evidence that black patients can present with more elevated baseline LDH and worse performance status [12].

Our findings show that in a real-world setting LDH appears to be the biomarker with most significant adverse prognostic value. Improved risk stratification for these patients may allow for consideration of individualized modifications in CAR T-cell therapy with use of maintenance therapy, administration of second infusion, addition of second anti-CD19 agent or CAR T-cell potentiating agents.

## Data Availability

Not applicable.

## Abbreviations

CAR: Anti-CD19 chimeric antigen receptor
DLBCL: Diffuse large B-cell lymphoma
LDH: Lactate dehydrogenase
OS: Overall survival
ECOG: Eastern Cooperative Oncology Group
R-IPI: Revised International Prognostic Index
CNS: Central Nervous System
CRS: Cytokine release syndrome
CARTOX: CAR T-Cell Therapy–Associated Toxicity score
ICANS: Immune effector cell-associated neurotoxicity syndrome
